# City of Chicago

**Published:** 1916-04

**Authors:** John Dill Robertson

**Affiliations:** Commissioner of Health


**City of Chicago.**

Department of Health replied as follows:

Your letter, asking for methods of terminating quarantine in contagious diseases, is received.

Diphtheria is the only disease which is terminated by bacteriologic evidence. We have requested hospitals to dismiss Typhoid cases only on absence of typhoid bacteriologic finding, and some of them are following the advice. It is feasible to terminate Typhoid fever in this way, but it means an adequate laboratory and more help in the field to secure specimens. It is the proper way to terminate Typhoid fever.

All other diseases quarantined, are terminated after a specified time period. This method can be used satisfactorily and with little danger of keeping up the quarantine too long. More danger of terminating too soon.

Under separate cover we are mailing you rules governing the quarantine of each disease. Yours very truly,

JOHN DILL ROBERTSON,
Commissioner of Health.

Rochester replied in brief that dismissal from quarantine based on negative bacteriologic evidence was in force for Diphtheria and Typhoid; and that in other cases, the time-limit method was satisfactory.

Owing to the miscarriage of blanks in the mails, we can report for Buffalo only the verbal information that diphtheria is the only disease released from quarantine on negative bacteriologic evidence.

